# 3D Printing Aids Acetabular Reconstruction in Complex Revision Hip Arthroplasty

**DOI:** 10.1155/2017/8925050

**Published:** 2017-01-10

**Authors:** Andrew J. Hughes, Cathal DeBuitleir, Philip Soden, Brian O'Donnchadha, Anthony Tansey, Ali Abdulkarim, Colm McMahon, Conor J. Hurson

**Affiliations:** ^1^Department of Orthopaedic Surgery, St. Vincent's University Hospital, Dublin 4, Ireland; ^2^Department of Mechanical Engineering, Institute of Technology Tallaght, Dublin 24, Ireland; ^3^Department of Orthopaedic Surgery, Cappagh National Orthopaedic Hospital, Dublin 11, Ireland; ^4^Department of Radiology, St. Vincent's University Hospital, Dublin 4, Ireland

## Abstract

Revision hip arthroplasty requires comprehensive appreciation of abnormal bony anatomy. Advances in radiology and manufacturing technology have made three-dimensional (3D) representation of osseous anatomy obtainable, which provide visual and tactile feedback. Such life-size 3D models were manufactured from computed tomography scans of three hip joints in two patients. The first patient had undergone multiple previous hip arthroplasties for bilateral hip infections, resulting in right-sided pelvic discontinuity and a severe left-sided posterosuperior acetabular deficiency. The second patient had a first-stage revision for infection and recurrent dislocations. Specific metal reduction protocols were used to reduce artefact. The images were imported into Materialise MIMICS 14.12®. The models were manufactured using selective laser sintering. Accurate templating was performed preoperatively. Acetabular cup, augment, buttress, and cage sizes were trialled using the models, before being adjusted, and resterilised, enhancing the preoperative decision-making process. Screw trajectory simulation was carried out, reducing the risk of neurovascular injury. With 3D printing technology, complex pelvic deformities were better evaluated and treated with improved precision. Life-size models allowed accurate surgical simulation, thus improving anatomical appreciation and preoperative planning. The accuracy and cost-effectiveness of the technique should prove invaluable as a tool to aid clinical practice.

## 1. Introduction

Total hip arthroplasty (THA) is one of the most successful and cost-effective interventions in medicine today, providing reliable pain relief and functional improvement to those with osteoarthritis or inflammatory arthritis of the hip [1, 2]. 90–95% of total hip replacement (THR) prostheses survive for at least 10 years, and there is an increasing demand within our population for such an intervention due to rising life expectancy among the ageing cohort with degenerative joint disease [3, 4]. Revision hip arthroplasty is indicated when a primary THR fails due to a variety of reasons, such as aseptic loosening (50%), instability (16%), infection (15%), debilitating pain, periprosthetic fractures, or component failure [5]. This complicated articular reconstructive procedure requires a comprehensive understanding of the abnormal bony anatomy. Surgeons must be able to appreciate areas of bony insufficiency, deficiency, and discontinuity, in order to conceptualise complex corrective reconstructions.

Conventional diagnostic imaging techniques provide only two-dimensional (2D) images of these deformities [6]. Orthopaedic surgeons develop experience in interpreting such 2D images when devising their operative strategies. More recently, advances in radiology combined with advances in computer and manufacturing technology have made the three-dimensional (3D) representation of anatomic structures relatively easily obtainable [7, 8]. Such images can be rotated and viewed from various angles, improving anatomical appreciation, but they must still be viewed on a flat 2D computer screen. With the use of modern rapid prototyping techniques, 3D models of actual osseous anatomy can be manufactured from these 3D reconstructed images [9, 10].

Rapid prototyping, or 3D printing, is a term used to describe a group of techniques used to quickly fabricate a scale model of a physical part or assembly using three-dimensional computer aided design (CAD) data. The origins of this technique can be traced back to the 1960s when Professor Herbert Voelcker described theories and algorithms for 3D model fabrication. Carl Deckard developed a technique to bind metal powders to create a 3D model in the University of Texas in 1987, before Charles Hull patented the first 3D printer in 1988 in California [8, 11]. Rapid prototyping has been used in the medical industry since the early 2000s, initially in the production of dental implants and patient-specific prostheses [12]. Since then, the use of 3D printing in the field of medicine and surgery has been rapidly expanding to include the development of soft tissue, organs, blood vessels, implants, and anatomical models [13]. Also within orthopaedic surgery, 3D printed models have been shown to improve the preoperative understanding of complicated structures in neurosurgery, liver transplant surgery, and vascular aortic surgery [11, 12, 14].

Revision hip arthroplasty is one of the most complex orthopaedic disciplines. Each case provides the surgeon with a challenge specific to the patient's unique anatomy. Often, 3D images are studied closely, but, as mentioned above, appreciation of the abnormality in question may not always be obtained on a 2D screen. The individual variances of the human body make the use of 3D printed models a valuable asset to surgeons when preparing for a complex procedure [14]. A 3D printed model provides visual and tactile reproduction of the deficient pelvic bony anatomy. This enables an improved understanding of the anatomy prior to surgery and facilitates enhanced preoperative planning [15].

## 2. Methods and Results

Life-size 3D models were manufactured from the computed tomography (CT) scans of two patients with complex acetabular defects waiting for second-stage THRs.

### 2.1. Case  1

**Figure 1:**
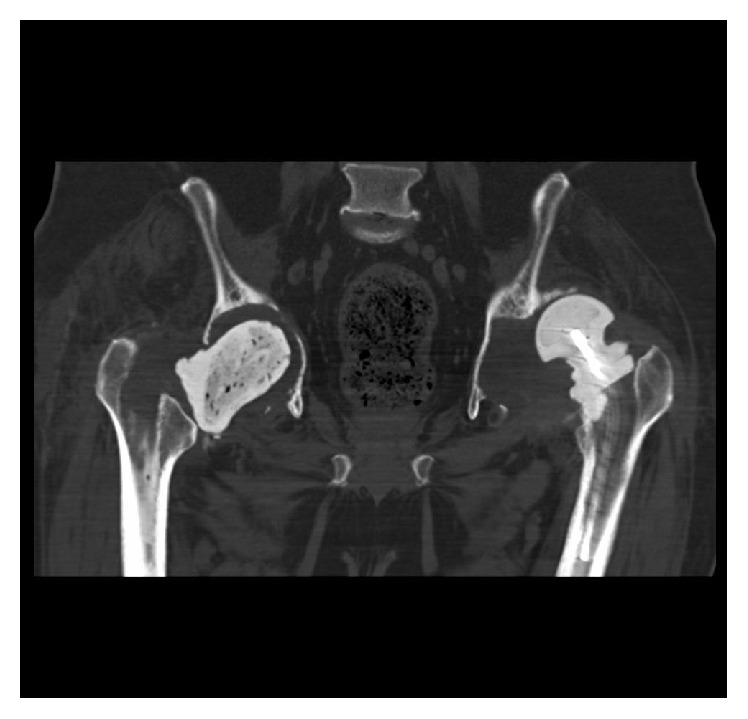
CT scan showing bilateral first-stage revision THR prostheses with right-sided pelvic discontinuity and a severe left-sided posterosuperior acetabular deficiency.

The first patient had a background of multiple bilateral hip arthroplasties for what was thought to have been aseptic loosening. Surgical intervention to date, however, had resulted in minimal symptomatic relief. Bilateral hip aspirations grew* Staphylococcus epidermidis* on enriched cultures. Bilateral first-stage hip revisions were subsequently performed, with bilateral antibiotic-coated spacers inserted. The postoperative CT scan of the pelvis showed right-sided pelvic discontinuity and a severe left-sided posterosuperior acetabular deficiency (see Figure 1). A six-week course of intravenous vancomycin and rifampicin was completed, as per the recommendation of the hospital's microbiology department.

### 2.2. Case  2

**Figure 2:**
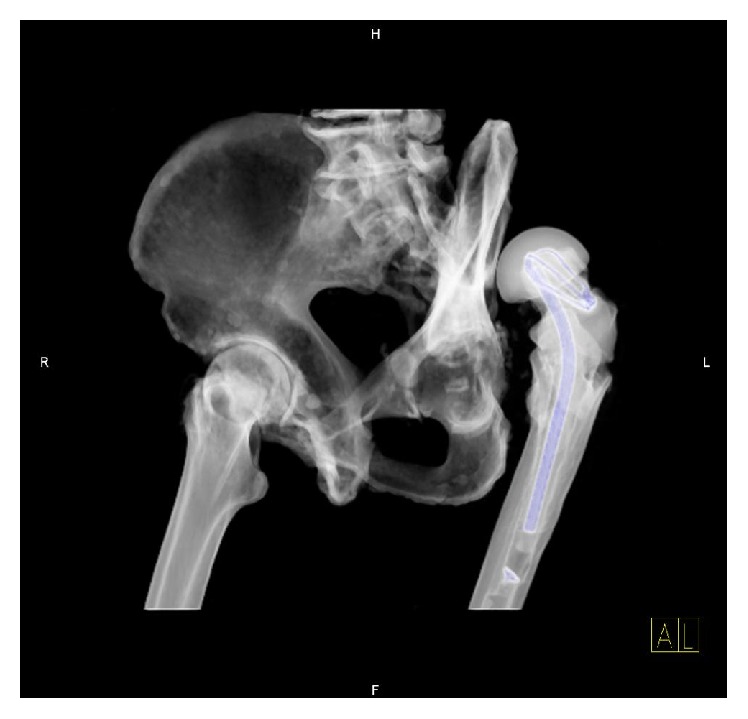
3D CT reconstruction showing a dislocated left-sided THR secondary to a posterior acetabular wall deficiency.

The second patient had undergone a first-stage hip revision, after preoperative aspiration confirmed an indolent* Staphylococcus epidermidis* periprosthetic joint infection, which had provoked multiple THR dislocations. Similar to the above, an antibiotic-coated spacer was inserted. A left-sided posterior acetabular wall deficiency was noted on the postoperative CT pelvis that was carried out (see Figure 2). Six weeks of intravenous daptomycin therapy was completed on consultation with the microbiology department. Both patients were listed for elective second-stage THRs as mentioned.

### 2.3. Rapid Prototyping

**Figure 3:**
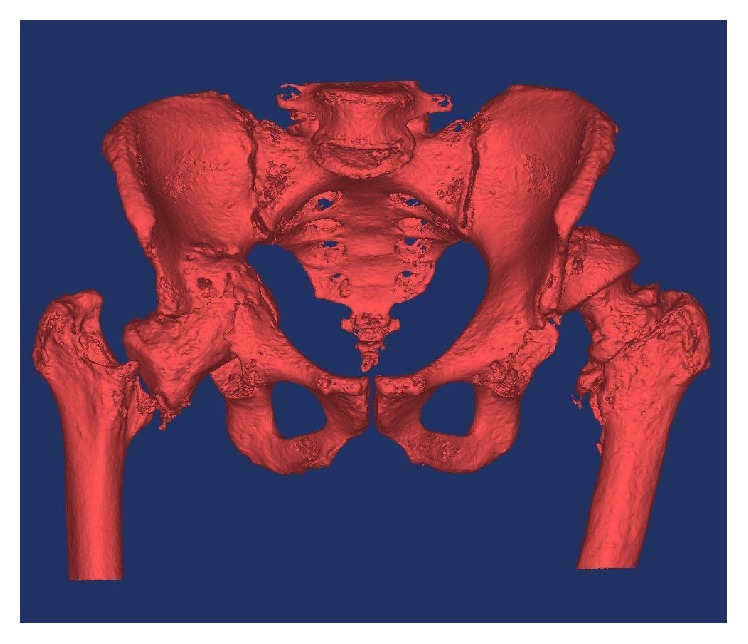
3D pelvic image created from the CT scan using Materialise MIMICS 14.12.

**Figure 4:**
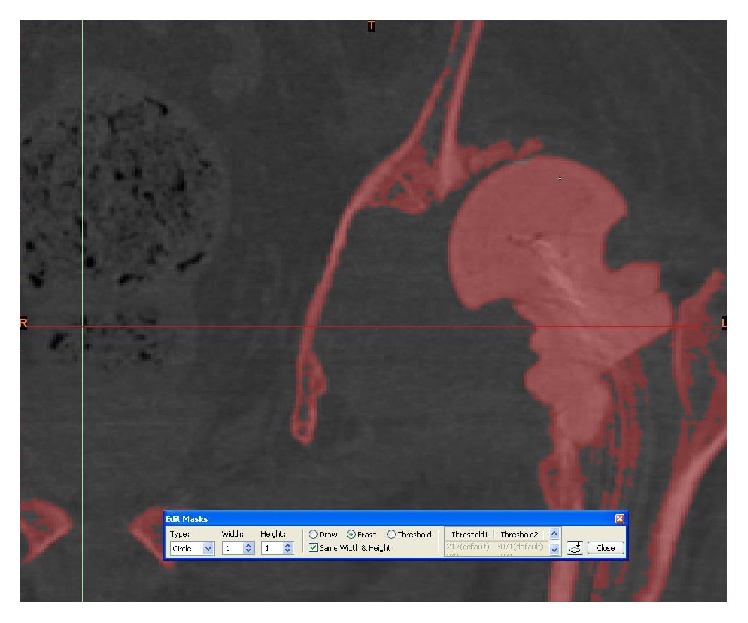
Segmenting the femur from the acetabulum using Materialise MIMICS 14.12.

**Figure 5:**
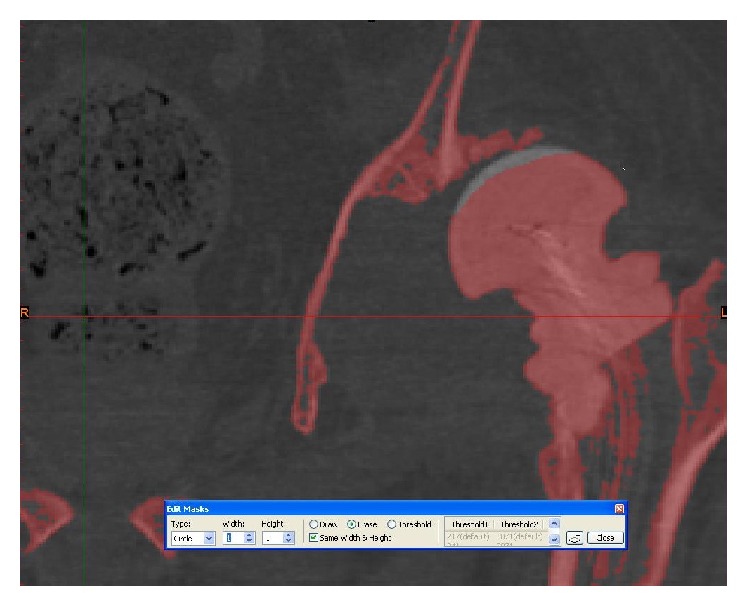
Segmenting the femur from the acetabulum using Materialise MIMICS 14.12.

**Figure 6:**
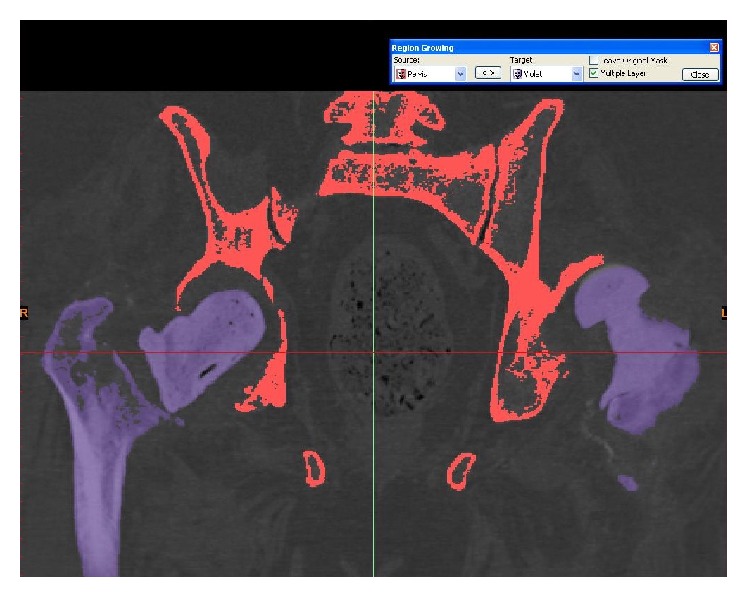
Thresholding of the pelvis and femoral bones.

**Figure 7:**
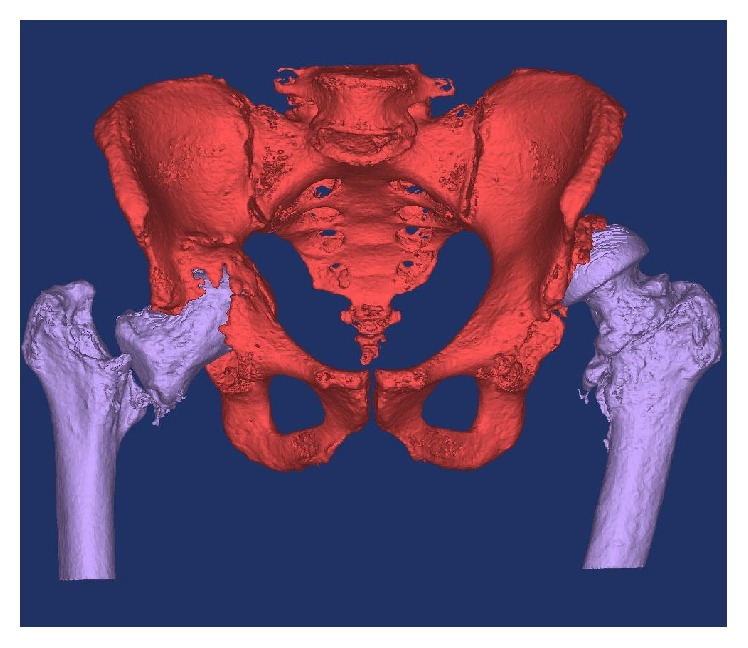
Thresholding and segmenting (deleting) the femoral bones from the pelvis.

**Figure 8:**
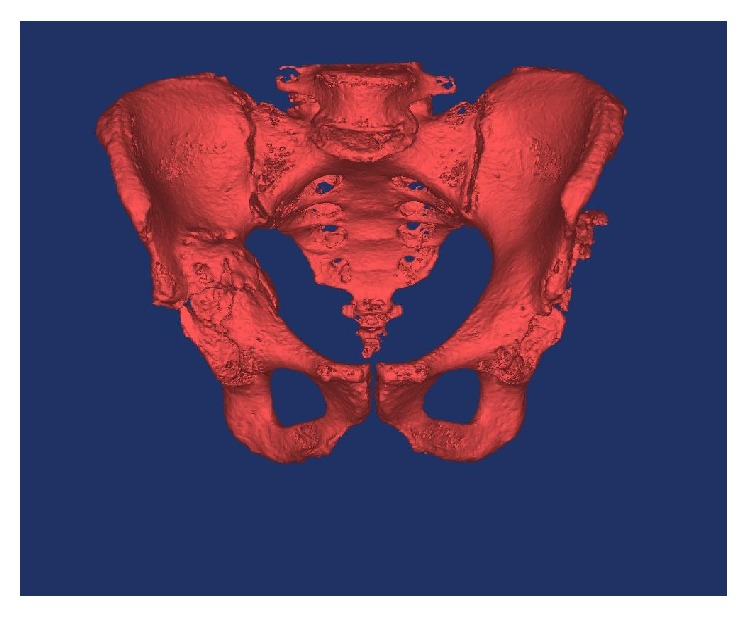
Thresholding and segmenting (deleting) the femoral bones from the pelvis.

Specific metal reduction protocols were used to reduce artefact on the two mentioned CT scans, with the slice thickness set to 1 mm to improve the image quality. The CT scans obtained were converted to DIACOM images and were then imported into Materialise MIMICS 14.12, medical imaging processing software, in the Mechanical Engineering Department of the Institute of Technology Tallaght, in Dublin. Image thresholding was performed, which allowed for bone to be differentiated from the surrounding soft tissue based on bone and soft tissue densities on the CT scan (see Figure 3). Using the region growing process, both femurs were digitally segmented from their corresponding pelvis by deleting the pixels that resulted in bony contact (see Figures 4, 5, and 6). The pelvis was isolated once both femurs were erased (see Figures 7 and 8). A 3D image of the isolated anatomy of interest was created on MIMICS. The imported file was saved in the  .STL (stereolithographic) format which allows instructions related to the shape, thickness, and texture of the 3D image to be communicated to the 3D printer [8, 11]. The two models were manufactured using the rapid prototyping process, selective laser scintigraphy (SLS). SLS is one of many 3D printing methods, which builds the part in question via successive nylon powder layers as the substrate. The powder is selectively fused together in corresponding cross sections through the use of a programmed carbon dioxide laser beam. The use of fine nylon as the powder medium has been reported to enhance the accuracy of this technique. This allowed for two detailed models to be manufactured [16].

**Figure 9:**
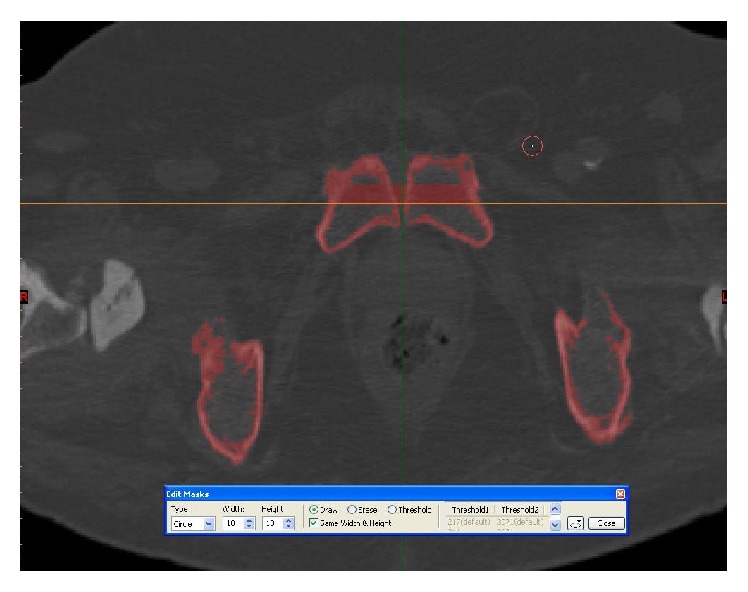
Support bars were drawn across the symphysis pubis and sacroiliac joints to avoid separation on printing the model.

**Figure 10:**
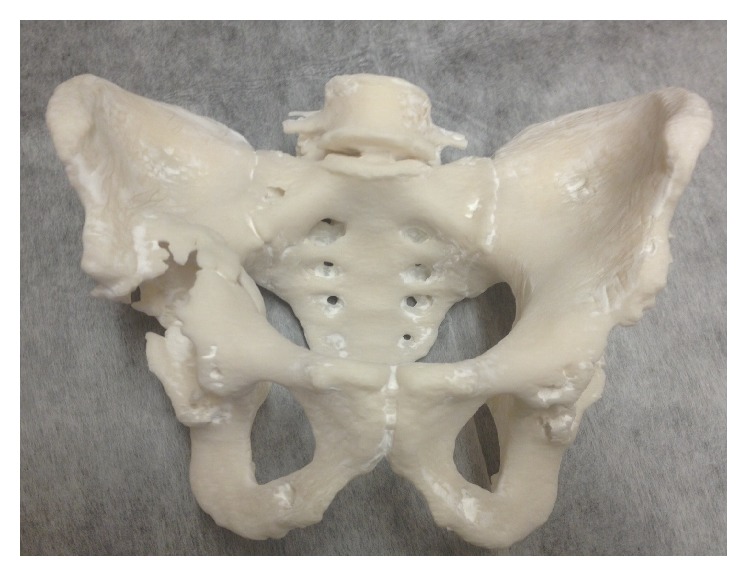
A life-size 3D printed model of the first patient's pelvis, providing the surgeon with visual and tactile appreciation of the defects in situ (note: these figures in print journal are two-dimensional, thus limiting the true demonstration of the value of 3D printed models in providing an accurate understanding and representation of the complex deformities and corrective reconstructive techniques).

The first patient, with bilateral hip spacers in situ, required a full pelvic model to be constructed. The second patient needed only that of a hemipelvis. The larger pelvic model contained minimal contact at a number of locations, in particular that of the pubic symphysis and both sacroiliac joints. Support bars were drawn across these articular surfaces, using MIMICS, so as to provide stabilisation and to prevent the model from collapsing once it was printed (see Figures 9 and 10).

### 2.4. 3D Models

**Figure 11:**
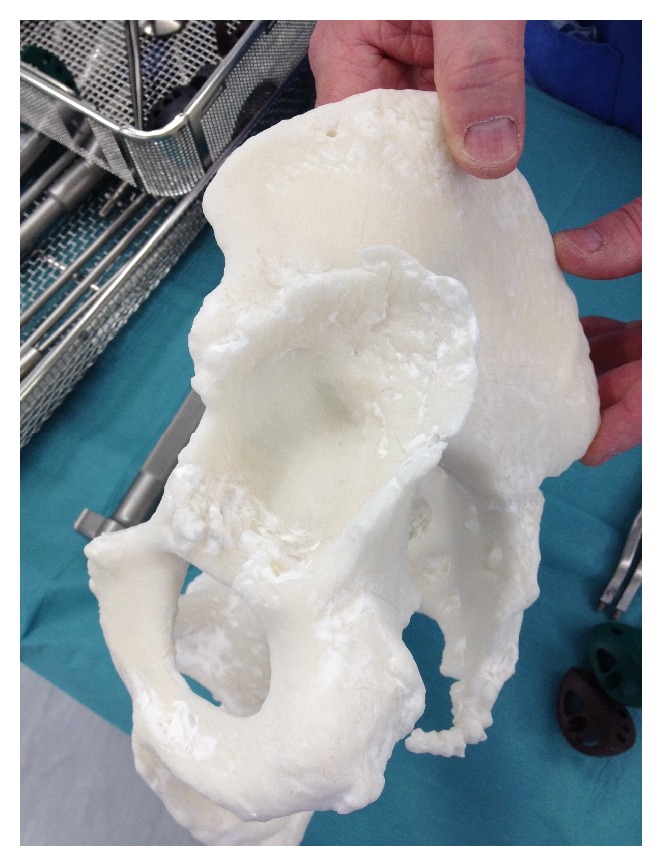
Examining the left acetabulum showing a posterosuperior deficiency. The right acetabulum shows significant central discontinuation due to bone loss (note: these figures in print journal are two-dimensional, thus limiting the true demonstration of the value of 3D printed models in providing an accurate understanding and representation of the complex deformities and corrective reconstructive techniques).

**Figure 12:**
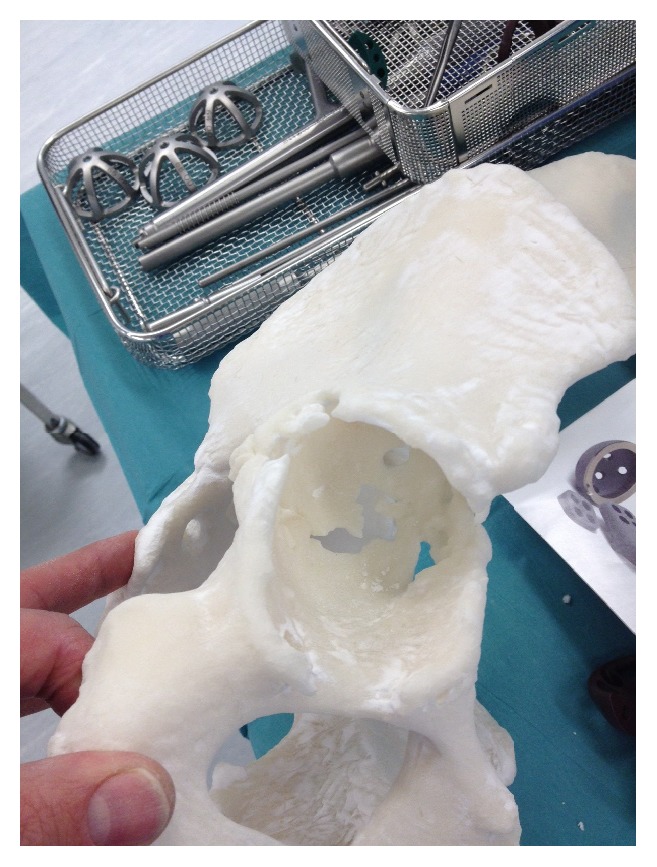
Examining the left acetabulum showing a posterosuperior deficiency. The right acetabulum shows significant central discontinuation due to bone loss (note: these figures in print journal are two-dimensional, thus limiting the true demonstration of the value of 3D printed models in providing an accurate understanding and representation of the complex deformities and corrective reconstructive techniques).

**Figure 13:**
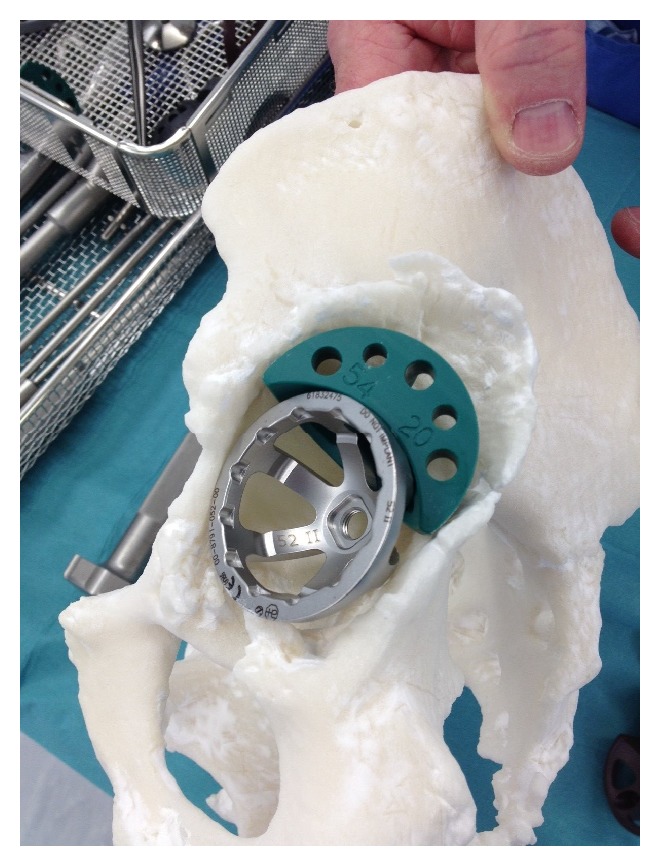
Preoperative templating, implant sizing, and surgical stimulation (note: these figures in print journal are two-dimensional, thus limiting the true demonstration of the value of 3D printed models in providing an accurate understanding and representation of the complex deformities and corrective reconstructive techniques).

**Figure 14:**
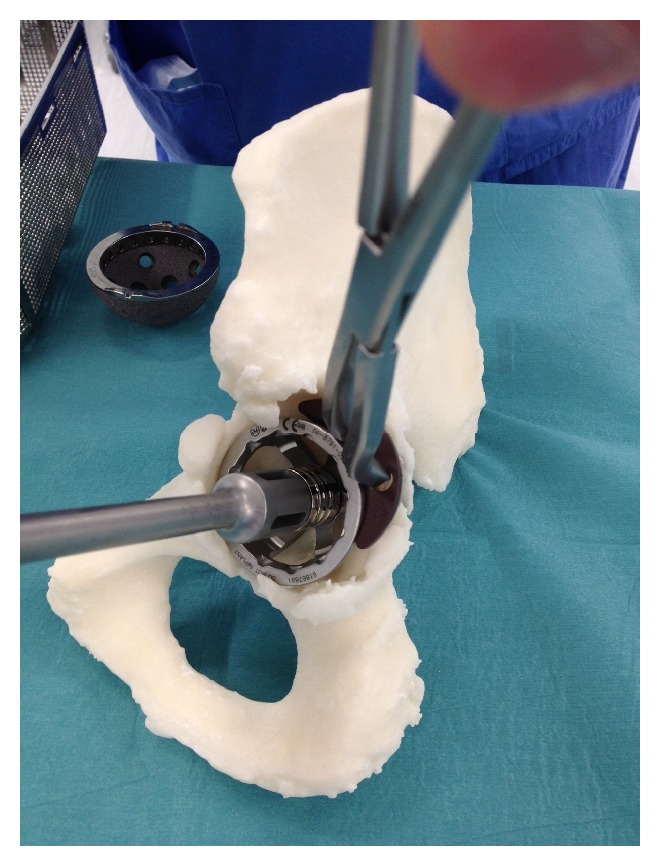
Preoperative templating, implant sizing, and surgical stimulation (note: these figures in print journal are two-dimensional, thus limiting the true demonstration of the value of 3D printed models in providing an accurate understanding and representation of the complex deformities and corrective reconstructive techniques).

**Figure 15:**
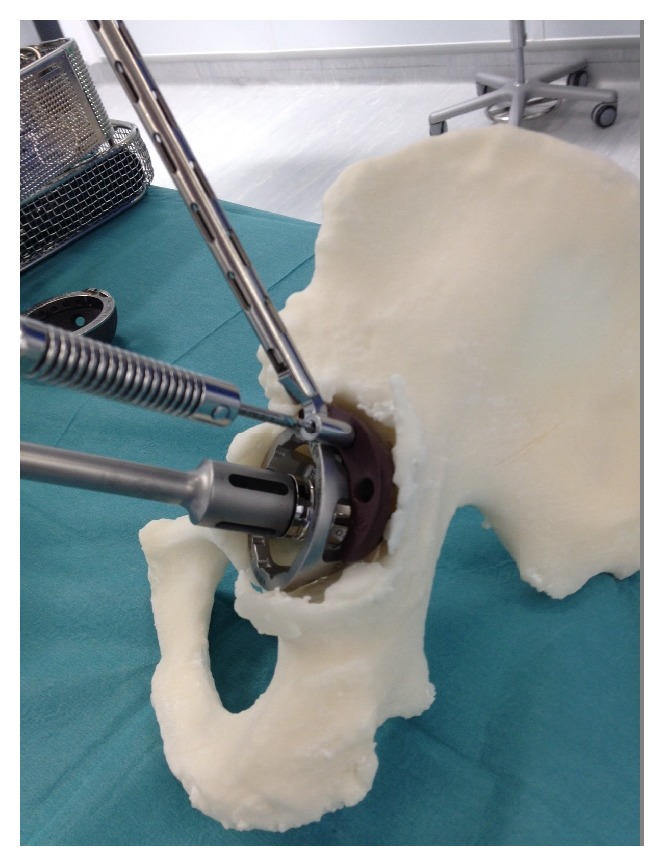
Preoperative templating, implant sizing, and surgical stimulation (note: these figures in print journal are two-dimensional, thus limiting the true demonstration of the value of 3D printed models in providing an accurate understanding and representation of the complex deformities and corrective reconstructive techniques).

The two 3D printed models provided the surgeon with visual and tactile appreciation of the three complex, irregular acetabula in question (see Figures 11 and 12). The two models cost $1450 ($400 and $1050) and were printed within twelve hours. Given the complexity of each case, these pelvic models allowed a life-size anatomical representation of the operative field to be closely examined. The operating surgeon described being able to identify and classify areas of bony deficiency. Following this, the team were able to plan and simulate a safe, successful surgical strategy. Templating was carried out by the surgeon in the weeks prior to surgery, and the implants were chosen accordingly. Acetabular cup, augment, and buttress sizes, as well as cage dimensions, were selected and trialled in advance. The malleable cage template was adjusted according to the contours of the model representing the patient's pelvic anatomy. This allowed prebending of the actual prosthesis prior to implantation before it was subsequently resterilised (see Figures 13, 14, and 15). The models were durable to a degree that allowed preoperative surgical simulation of drill trajectory and screw positioning in cortical bone.

**Figure 16:**
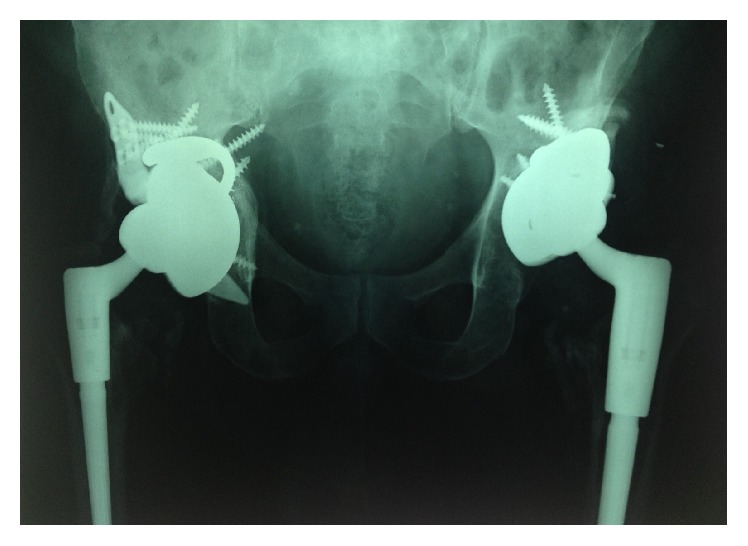
Postoperative anteroposterior pelvic plain film radiographs showing second-stage THR revisions in situ.

**Figure 17:**
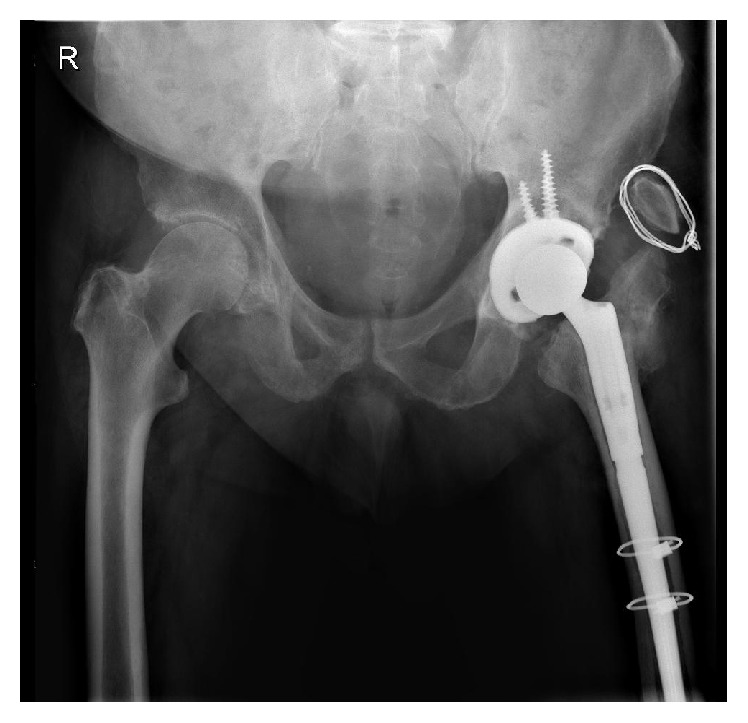
Postoperative anteroposterior pelvic plain film radiographs showing second-stage THR revisions in situ.

The use of preoperative templating using these 3D models reduced operative time and blood loss and improved intraoperative surgical decision-making. Screw trajectory simulation was carried out on the models, allowing for improved accuracy and thus reducing the chance of intraoperative neurovascular injury. Screw position simulation allowed for the best use of available bone stock and helped ensure the best construct stability. Both patients underwent second-stage hip revision in Cappagh National Orthopaedic Hospital on an elective basis (see Figures 16 and 17). The outcome in both cases was satisfactory, with rehabilitation completed in a designated unit on discharge. Antibiotic therapy postoperatively was continued and completed as per the continued input of the microbiology department. Both patients are mobilising more than three years postoperatively with no signs of infection or loosening. The surgeon described overall satisfaction with the life-size pelvic models. Improved anatomical classification, preoperative surgical planning, and intraoperative accuracy resulted in a shorter, safer procedure with less perioperative morbidity and the efficient use of hospital resources.

## 3. Discussion

The complex anatomy of the pelvis and the acetabulum makes preoperative assessment of such abnormal bony deficiencies in revision hip arthroplasty notoriously difficult. With the use of 3D printing technology, pelvic deformities can be better evaluated by examining visual and tactile models of the patient's actual osseous anatomy. The internal structure is represented as a life-size 3D structure that can be held, rotated, and viewed by the operating team in advance of the procedure. As a result, complicated revision cases can be thoroughly evaluated and classified preoperatively, giving the surgeon an opportunity to treat the patient with improved surgical precision. Hurson et al. described the use of 3D models in assessing the acetabulum of 20 patients. In two of their cases, the initial surgical approach, having studied the patients' imaging, was altered based on further review of life-size anatomical 3D models [15].

The use of the models, combined with conventional imaging, has been shown to result in a greater understanding of abnormal bony anatomy, when compared to that of two- and three-dimensional imaging alone [11, 12]. 3D imaging enhances the understanding of surgeons and radiologists when assessing bony deformities; however, they must be viewed on 2D screens. As a result, the true benefits of 3D imaging are often lost by this limitation. Tactile models have been shown to improve diagnostic accuracy and interobserver agreement when assessing abnormal acetabular anatomy. The combined use of conventional imaging modalities and anatomical models improved the accuracy further of trainees assessing acetabula and aided in their education by serving as demonstrative tools [15].

Life-size models allow accurate surgical simulation, enabling preoperative cup, augment, and buttress sizing, as well as cage templating and screw trajectory optimisation. Similar to our experience, Won et al. showed that this technique can reduce the intraoperative complications as described above [17]. 3D models have also been described to be of significant use in acetabular surgery preoperatively when implants require contouring in three planes. Performing this in advance of surgery, combined with trialling the implant's positioning, reduces operative and anaesthetic times [8].

In addition to their use in revision hip arthroplasty, 3D models use has also been described in spinal, dental, and maxillofacial surgery. Across the varying surgical specialties, 3D models have been used, similar to our experience, to gain preoperative insight into a patient's anatomy [12]. From a teaching perspective, the use of 3D models has been employed as a tool in subjects such as anatomy, obstetrics, dentistry, and embryology [8].

With regard to surgically applied anatomy, 3D models have been shown to be more cost-effective when compared to cadavers. 3D models allow the trainer to demonstrate the presence of pathology, which is not the case in cadaveric training. Having said this, the latter is recognised as being very beneficial when it comes to anatomical teaching, just less so when it comes to surgical simulation [11, 18].

Patient-specific implants and prostheses can now be manufactured worldwide within 24 hours. In complex cases, it can prove difficult to fit patients with a suitable prosthesis, in particular for those undergoing extensive limb salvaging oncological procedures. In other disciplines such as dental and maxillofacial surgery and neurosurgery, it is often not possible to use a standardised implant. As a result, availing of 3D printing in unusual cases can produce a satisfactory result [12, 14, 18].

We described our experience of using rapid prototyping successfully in three complex revision hip arthroplasty cases in two patients. Whilst our results were encouraging, the number of documented cases in the literature remains relatively limited [19]. Our experience was one of satisfaction; however, there was no objective measurement of the benefit, and there was no comparison made with similar complex cases that did not use 3D printed models. The availability of 3D printing continues to improve and its cost continues to decline; however, barriers exist with regard to the regulation, safety, and security of the widespread use of 3D printing in surgery [19, 20].

## 4. Conclusion

Three complex second-stage hip revision cases were identified in two patients preoperatively. Rapid prototyping and 3D printing were used to produce life-size 3D models of three hips. Acetabular reconstruction was planned, trialled, and managed efficiently with improved surgical precision and reduced complications. The accuracy and cost-effectiveness of this technique in both cases as described were impressive and its increasing use should prove invaluable as a tool to aid clinical practice and education in the future.
